# Modeling Pine Caterpillar, *Dendrolimus spectabilis* (Lepidoptera: Lasiocampidae), Population Dynamics with a Stage-Structured Matrix Model Based on Field Observations

**DOI:** 10.3390/insects17010056

**Published:** 2026-01-01

**Authors:** Young-Kyu Park, Youngwoo Nam, Won Il Choi

**Affiliations:** 1 Korean Beneficial Insects Lab. Co., Ltd., Gokseong 57507, Republic of Korea; 2 Forest Entomology and Pathology Division, National Institute of Forest Science, Seoul 02455, Republic of Korea; orangmania99@korea.kr

**Keywords:** *Dendrolimus spectabilis*, forest pest, matrix model, population dynamics, sensitivity analysis

## Abstract

Population models offer insights into both theoretical and practical aspects of insect population dynamics. Among the models, stage-structured matrix models are used to describe the population dynamics of insects because the development of insects is by nature stage-structured. Field populations of the pine caterpillar, *Dendrolimus spectabilis* (Lepidoptera: Lasiocampidae) were monitored in a pine stand located in Dorak-ri, Cheongsan-myeon, Wando-gun, Jeollanam-do, from May 1998 to March 1999, and the pest density was measured as the number of larvae, pupae, or eggs at one-month intervals, excluding the winter season. Life tables and matrix models were constructed based on field observations, and the most vulnerable life stage was identified through sensitivity analysis. The density of the pine caterpillar (number per 1000 cm^2^ branch) was 7.9 on 8 May 1998, and subsequently decreased to 0.5 on 14 March 1999, showing a decreasing trend of caterpillar density. The population growth rate was 0.74, a decreasing trend. The most vulnerable stages were (1) the larvae immediately after hatching and (2) again during overwintering, probably due to indirect mortality caused by humid conditions and activities of natural enemies during winter. Given the significant damage caused by mature larvae in the spring and that the density of the caterpillar after overwintering typically remains stable, forest management requires that the pest density be monitored soon after overwintering to allow decisions about control measures to be taken. Our results showed that a matrix model is useful to describe the population dynamics of the pine caterpillar and to construct suitable management strategies.

## Simple Summary

The pine caterpillar (*Dendrolimus spectabilis*) is a major forest pest that defoliates pine trees in Korea. The moth population was monitored for one year at monthly intervals, excluding winter. The census data obtained was used to construct life tables and a stage-structured matrix model based on the number of eggs, larvae, and pupae per branch area. Sensitivity analysis revealed that newly hatched larvae and overwintered larvae were the most vulnerable stages, primarily influenced by abiotic factors such as rainfall and temperature. This modeling approach suggests that pest management should prioritize monitoring populations immediately after overwintering. Overall, the study demonstrates that matrix models are useful tools for understanding forest insect dynamics and for designing timely, stage-specific control strategies.

## 1. Introduction

Modeling of insect population dynamics provides valuable insights from both a theoretical and practical perspective because key drivers determining population density are identified, and population density is predicted by modeling approaches [1,2,3]. Among population models, age- or stage-structured models are appropriate to describe population dynamics of insects because insect development is fundamentally stage-structured [4,5,6,7]. Age or stage structured matrix models (called Leslie and Lefkovitch Models, respectively) have been proposed to describe population dynamics based on survival probability and fertility [5,6,7]. In case of plants and insects, describing individuals by developmental stage rather than chronological age is often more biologically meaningful [7]. Several methods to describe the effects of matrix elements (survival probability and fertility) on population growth rate were proposed through sensitivity analysis [4]. Until now, matrix models have been most often used in the areas of conservation biology and pest management. Using a matrix population model, Strömbom and Pandey [8] demonstrated that the combined application of natural enemies, including the egg parasitoid *Ooencyrtus kuvanae* (Howard) and the entomopathogenic fungus *Beauveria bassiana* (Bals.) Vuill. effectively reduced the population growth rate of the spotted lanternfly (*Lycorma delicatula* (White)). Impacts of extreme climate, including heat exposure and drought, on population growth of *Pieris napi* (Linnaeus), a common butterfly species in Mediterranean areas, were analyzed using a matrix population model [9].

Outbreaks of the pine caterpillar (*Dendrolimus spectabilis* (Butler)) have a recorded history of approximately 1000 years, with its first recorded outbreak being in 1100, as noted in the History of the Goryeo Dynasty (Goryeosa) [10,11]. This univoltine species damages Korean red pine (*Pinus densiflora* Siebold and Zucc) and Japanese black pine (*P. thumbergii* Parl) in the spring [12]. It pupates from July, whereupon adults lay their egg masses in August. First instar larvae hatch from eggs in August or early September, and most larvae overwinter as fifth instars [12]. Neonate larvae are vulnerable to heavy summer rains, which can wash small larvae off host plants [13] and increase in risk of fungal infections due to high humidity [14]. In addition, low winter temperatures can increase overwintering mortality, thereby reducing population density [15]. Due to the success of forest restoration, outbreaks of the pine caterpillar decreased dramatically starting in the 1970s, with outbreaks observed only locally in the interior of Korea [11,16]. However, areas defoliated by *Dendrolimus* spp. in China are expected to increase in the future due to more severe drought periods caused by climate change [1]. Moreover, local outbreaks of the pine caterpillar have periodically been observed in Korea since the 1980s [11]. Therefore, there is a need to better understand how climatic factors affect pine caterpillar population dynamics.

We used data from a field survey in southern South Korea to estimate the density of pine caterpillar eggs, larvae, and pupae inhabiting a stand of pine trees. The observed changes in population densities were used to construct a life table for one generation of *D. spectabilis*, and to develop and parameterize a stage-structured matrix model. By means of the model, we conducted a sensitivity analysis with the purpose of identifying the life stage(s) that had the strongest impact on the species’ population densities. Our study was carried out to improve the understanding of the pine caterpillar population dynamics and thereby provide a better theoretical foundation for developing effective management strategies of this pest.

## 2. Materials and Methods

### 2.1. Field Surveys of Pine Caterpillar

**Figure 1:**
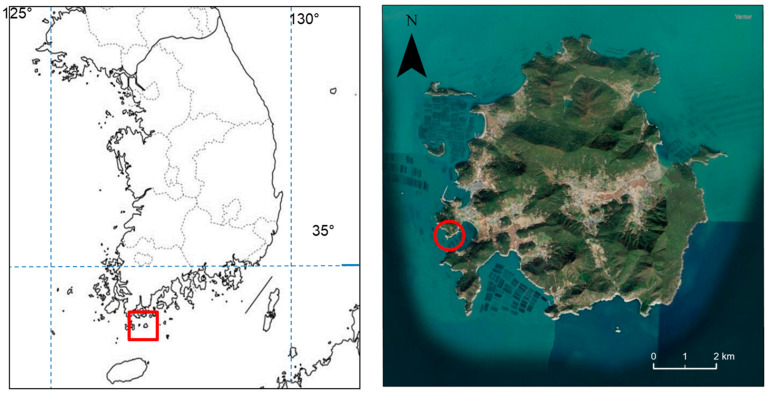
The location of the study site for the pine caterpillar in South Korea is marked with a red circle. The site is located near the village of Dorak-ri on the island of Cheongsan in Wando-gun County within Jeollanam province.

Density of pine caterpillar life stages was measured in a pine stand in Korea (Dorak-ri, Cheongsan-myeon, Wando-gun, Jeollanam-do). The study area was defined as a plot approximately 250 × 150 m (34°10′ N, 126°51′ E) (Figure 1). The stand consisted of young Korean red pine with an average tree height of approximately 1.7 m, located near the seashore. The annual average temperature in this region in the study years was 14.3 °C, and the annual precipitation was 1531.5 mm (https://data.kma.go.kr, accessed on 1 September 2025). Meteorological data were collected from a weather station located approximately 30 km to the northwest.

Pine caterpillar density was recorded from May 1998 to March 1999 at one-month intervals, excluding the winter season because it is the overwintering period of the caterpillar. Thirty trees spaced at least 5 m apart were randomly selected. From each tree, one branch was randomly sampled from the upper part of the canopy and one from the lower part. The number of pine caterpillar larvae, pupae, and eggs was counted, and the length and diameter of the sampled branches were measured. The number of eggs was first recorded in the field as egg masses, which were then brought to the laboratory for counting. Moth stage density was recorded as the number of larvae, pupae, or eggs per 1000 cm^2^ (length × diameter) of branch surface area. Sampled trees were randomly selected at every observation date. Densities of the pine caterpillar were expressed as mean ± standard error (SE). Concurrently, numbers of larvae that had been killed by parasitoids or entomopathogenic microorganisms were recorded, but without agent identification to the species level.

### 2.2. Life Table Construction

The life table of the pine caterpillar was constructed based on field observations. The moth life cycle was divided into six developmental stages: eggs (E), four larval instars (L1, L2, L3, L4), and pupae (P). Larval stages were defined as follows: L1 (autumn larvae soon after egg hatch), L2 (autumn larvae just before overwintering), L3 (early spring larvae after diapause), and L4 (late spring larvae).

The number of each stage (*n_x_*) was estimated per 1000 cm^2^ of branches, except for the adult stage, because adult density could not be directly observed in the field. Instead, fecundity of the moth was estimated from the number of eggs observed per 1000 cm^2^ of branch surface area in the field, as an empirical indicator of realized egg production. The probability of survival of each stage (*g_x_*) was calculated as *g_x_* = *n_x_*/*n_x−_*_1_*,* and the proportion of individuals surviving relative to the initial density (*l_x_*) was calculated as *l_x_* = *n_x_*/*n_a_*, where *n_a_* represents the initial density of the pine caterpillar, which in this case refers to the density of eggs.

### 2.3. Matrix Model Construction

**Figure 2:**
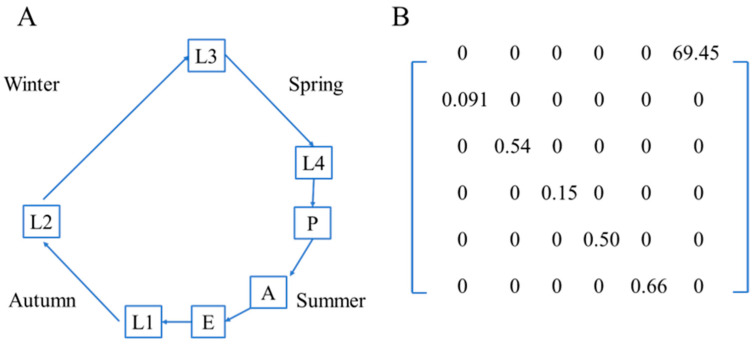
Life cycle of the pine caterpillar (**A**) and Leslie Matrix of the pine caterpillar estimated from field observations (**B**).

We constructed a stage-structured matrix model to describe the life cycle of *D. spectabilis*. The model was formulated as an annual matrix model, in which one projection step represents one year (one generation of the species). A 6 × 6 Leslie matrix is constructed based on field observations and the species’ life cycle (Figure 2A). The survival probability of each stage (*g_x_*) represented the stage transition probabilities. Egg density was considered as realized fecundity. The population growth rate was estimated as the dominant eigenvalue of the Leslie matrix.

Matrix characteristics, including the population growth rate, stable age structure, and sensitivity, were analyzed using the R version 4.3.1. package popbio [17,18]. Sensitivity represented the effect of each matrix element (stage-specific survival and fecundity) on the population growth rate. The effect of each matrix element on the population growth rate was evaluated by varying the focal parameter while keeping the other parameters constant. Survival rates at each stage were increased by increments from 0.1 to 1.0 at intervals of 0.1, and fecundity was varied with increments of 10 between 50 and 500.

## 3. Results

**Figure 3:**
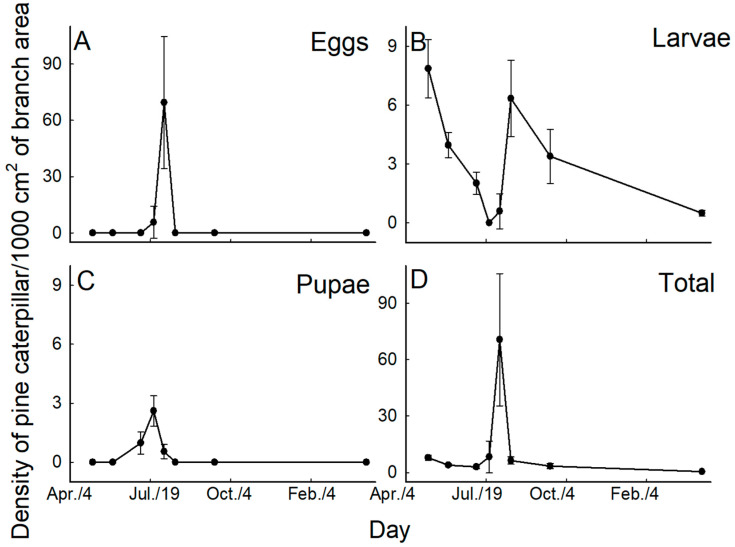
Sequential changes in the density of pine caterpillar eggs (**A**), larvae (**B**), pupae (**C**), and the total of all stages except adults (**D**). The density of caterpillars is expressed as the number of individuals per 1000 cm^2^ of branch surface.

The density of the pine caterpillar larvae per 1000 cm^2^ branch was 7.9 ± 1.5 on 8 May 1998 and gradually decreased to 2.0 ± 0.6 by 7 July 1998. After egg hatch, larval density of the next generation was 6.3 ± 1.9 on 19 August and subsequently decreased to 0.5 ± 0.2 by 14 March 1999 (Figure 3A). The pupal and egg stages were observed only in summer, with the highest pupal density (2.6 ± 0.8) being recorded on 23 July, while the highest egg density (69.5 ± 35.1) occurred on August 5 (Figure 3B,C).

Larval mortality due to entomopathogenic microorganisms, such as fungi or viruses, in early spring was 13.2% after overwintering. After the first survey and before pupation, 15.8% of the larvae were killed by parasitoids, entomopathogenic microorganisms, or birds. Direct causes of the pupal mortality were not observed in July, and mortality agents of autumn larvae were not observed in the field.

The life table reflected a similar declining trend, with only 0.2% of the initial population surviving to the final stage (Table 1). The lowest and highest stage-specific survival probabilities were 0.091 at L1 and 0.66 at the pupal stage, respectively (Table 1, Figure 2B). Realized fecundity based on field observation was estimated as 69.5 ± 35.1, although the number of eggs per egg mass ranged from 139 to 265, with a mean of 169.0 ± 16.9.

**Figure 4:**
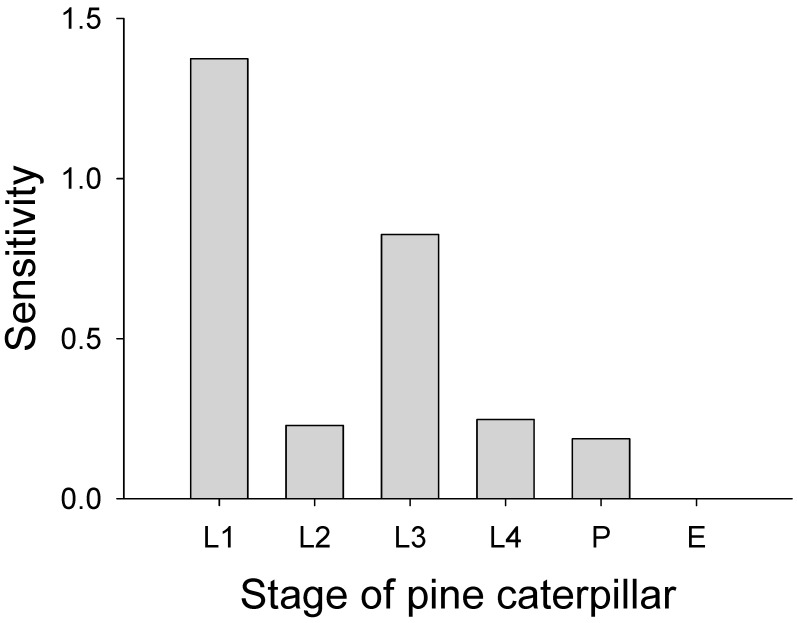
Sensitivities of each stage of the pine caterpillar are estimated from Lesile matrix models. E: egg stage, L1: autumn larvae after egg hatch, L2: autumn larvae before overwintering, L3: early spring larvae after diapause, L4: late spring larvae, and P: pupal stage.

The population growth rate estimated from the matrix model was 0.74, indicating a consistent population decline. The stable stage structure was L1: 0.80, L2: 0.097, L3: 0.071, L4: 0.014, P: 0.0096, and A: 0.0085. Sensitivity analysis indicated that L1 and L3 were the most sensitive stages (Figure 4).

**Figure 5:**
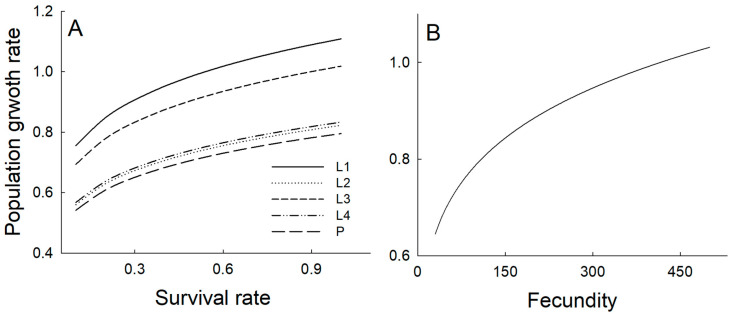
The effect of survival probability (**A**) and fecundity (**B**) in matrix models on the population growth rate was evaluated by varying the focal parameter while keeping others constant. Survival rates were increased incrementally from 0.1 to 1.0 at intervals of 0.1, and fecundity values, which ranged from 50 to 500 eggs, were increased at increments of 10. L1: autumn larvae after egg hatch, L2: autumn larvae before overwintering, L3: early spring larvae after diapause, L4: late spring larvae, and P: pupal stage.

Increases in the survival rate of each developmental stage variably influenced the population growth rate. In sensitivity analysis, increasing the survival probability of L1 or L3 stages resulted in a predicted increase in the population growth rate to values exceeding 1 (i.e., population growth). However, increases in survival probabilities in the L2, L4, and pupal stages elevated the population growth rate but did not bring it above unity. Likewise, fecundity increases raised the population growth rate above one only when fecundity exceeded 420 (Figure 5).

## 4. Discussion

Our results show that Leslie matrix modeling of the pine caterpillar based on field observations was useful to understand population dynamics of the moth because the most sensitive stages affecting population growth rate were identified through sensitivity analysis. Survival probability after egg hatch of either young (L1) or overwintering (L3) larvae strongly affected the population growth rate of the pest, suggesting these two stages were the most sensitive stages. Increases in survival probabilities during the autumn and spring larval stages, as well as the pupal stage, were insufficient to elevate the population growth rate to unity when the survival probabilities of L1 larvae after egg hatch (i.e., young larvae) and overwintering larvae (L3) were held constant. The population growth rate only reached unity when the realized fecundity was exceptionally high.

It is likely that the factors influencing the two sensitive stages are density-independent, particularly in relation to climate variables. The population density of the young pine caterpillar larvae right after egg hatch was strongly affected by precipitation in August because heavy precipitation increases the mortality of young larvae in South Korea [19]. In Japan, 70% to 80% of the first and second instar larvae died after heavy precipitation [20]. These losses are direct mortality caused by heavy precipitation washing larvae off host plants [13] and, indirectly, by rain lowering egg hatch and causing higher larval mortality from disease due to higher humidity [14]. In our study, egg masses and early-instar larvae were predominantly recorded on 5 August and 19 August. During the corresponding period (1–19 August), cumulative precipitation amounted to 73.2 mm (mean 6.7 mm), with precipitation on 11 of 19 days. This suggests that the decline of the pine caterpillar density that we observed was probably caused indirectly, rather than by washing effects. In late September (from 20–30 September), the population experienced heavy precipitation (405.7 mm, which was 22.2% of annual precipitation), and the density of older larvae decreased by approximately 46%. However, the decline had no substantial impact on the overall population growth rate.

The second sensitive stage was the decline of larval density after overwintering., Mortality of the Chinese pine caterpillar, *Dendrolimus tabulaeformis* Tsai et Liu, a close relative to *D. spectabilis* and with similar geographical distributions [21], was caused by temperatures below −15 °C [22]. Considering that the average temperature in January 1999 was 3.4 °C and the daily minimum temperature reached −5.2 °C, the decline in density of the larvae in our study area was unlikely to reflect direct mortality from low temperatures. Similarly, records of the pine-tree lappet moth, *Dendrolimus pini* (Linnaeus), increased in warm winters, including late autumn and early spring, through changes in host plant condition, causing faster development of larvae, as well as greater vitality and feeding activity of the larvae [23]. Additionally, the activity of entomophagous microorganisms also caused part of the decline in larval numbers, with 13.2% of larvae being infected with pathogens in early spring. Overwintering mortality of Siberian silkworm caterpillars (*Dendrolimus sibiricius* Chetverikov), ranging from 23.5 to 38.7%, was also due to microorganisms [24]. Overwintering mortality due to entomopathogenes in *D. pini* was also observed in European countries, including Germany, Lithuania, and Croatia, with the highest mortality being 98.4% (in Croatia) [23].

The two most sensitive stages identified through matrix modeling in our study were mainly affected by density-independent factors, including precipitation and temperature. Of course, these density-independent factors affected the biological traits of the caterpillar and the activity of its natural enemies. One of the major density-dependent factors, mortality by natural enemies, was observed to be relatively low compared to the overall mortality of the two most sensitive stages.

The influence of weather on the population growth of the pine caterpillar suggests that climate change does affect the population dynamics of the moth. Increases in winter temperatures and longer periods of drought are expected to occur due to climate change [25,26]. Increased winter temperatures are likely to be favorable for the population growth of the pine caterpillar in Korea. However, the impact of drought on the pest will likely depend on the pattern of precipitation. Climate change may influence both the amount and the timing of precipitation and likely increase the frequency of intense precipitation. Temporal synchrony between intense precipitation and the younger larval stage, vulnerable to heavy rain, would be unfavorable for the population growth of the pine caterpillar. In Germany, high autumn temperatures were found to be unfavorable for the population growth of *D. pini* [3,27]. Furthermore, increases in temperature due to climate change may cause a shift in the voltinism of pine caterpillar from univoltine to bivoltine in Korea, which may be unfavorable to the pine caterpillar population because adults of the second generation were generally smaller than those of the first generation, likely due to heat stress in summer and the consumption of hardened, mature needles [16].

We identified the most sensitive life stages of the pine caterpillar, which leads to recommended management strategies. The two most sensitive stages were identified to be first instar larvae appearing shortly after egg hatch and fifth instars occurring after overwintering. Fluctuations in survival of other life stages had relatively little effect on population growth. Given that most damage is caused by mature larvae after their overwintering, we recommend assessing the density of this stage, and in case the density exceeds the economic threshold, initiating control measures against the pest.

## 5. Conclusions

Our results show the usefulness of matrix models in analyzing the population dynamics of forest insects and in developing appropriate management strategies. By identifying the most vulnerable stages, we were able to determine the optimal timing for monitoring and control. This provides a clear example of how matrix models can be effectively applied in forest pest management. Furthermore, the development of models that incorporate interactions between pests and their natural enemies would further enhance our understanding of forest insect population dynamics and improve the effectiveness of biological control strategies. Therefore, future efforts should focus on advancing such integrative modeling approaches.

## Figures and Tables

**Table 1:** The number of live pine caterpillars (*Dendrolimus spectabilis* per 1000 cm^2^ of branch surface) at stage (*n_x_*), the survival probability of surviving from stage *x* − 1 to stage *x* (*g_x_*), and the probability of surviving from egg to stage *x* (*l_x_*), were estimated from field sampling in 1998 to 1999.

Stage	*n_x_*	*g_x_*	*l_x_*
Eggs (E)	69.45		
Larvae in autumn (L1)	6.33	0.091	0.09
Larvae before diapause (L2)	3.39	0.54	0.05
Larvae after diapause (L3)	0.49	0.15	0.007
Larvae in spring (L4)	0.25	0.50	0.004
Pupae (P)	0.16	0.66	0.002

*n_x_*: the number of live pine caterpillars per 1000 cm^2^ pine branch of stage *x*; *g_x_*: surviving probability in stage *x*; *l_x_*: proportion of survivors relative to the initial density.

## Data Availability

The original contributions presented in this study are included in the article. Further inquiries can be directed to the corresponding author.
